# Wastewater-based reproduction rates for epidemic curve reconstruction

**DOI:** 10.1093/biostatistics/kxaf033

**Published:** 2025-10-17

**Authors:** Emily Somerset, Justin J Slater, Patrick E Brown

**Affiliations:** Department of Statistical Sciences, University of Toronto, 700 University Avenue, Toronto, Ontario, M5G 1Z5, Canada; Department of Mathematics and Statistics, University of Guelph, 50 Stone Road East, Guelph, Ontario, N1G 2W1, Canada; Department of Statistical Sciences, University of Toronto, 700 University Avenue, Toronto, Ontario, M5G 1Z5, Canada

**Keywords:** Gaussian processes, wastewater-based epidemiology, infectious disease modeling, Bayesian hierarchical model, modular Bayesian inference

## Abstract

We introduce a hierarchical Bayesian framework for reconstructing epidemic curves using under-reported case counts and wastewater data. Our approach models wastewater signals as differentiable Gaussian processes, enabling inference on their relative growth rates, which are used to define a wastewater-based reproduction rate. These estimates are incorporated into a binomially thinned Poisson autoregressive model for case counts using a modular inference strategy. We apply this framework to reconstruct the Covid-19 epidemic curve in Toronto, validating our model through out-of-sample forecasts and comparisons with independent serosurvey-based cumulative incidence estimates. We also apply the framework to New Zealand’s Covid-19 data to reconstruct its epidemic curve and demonstrate improvements over an existing joint model for wastewater and case data. A key advantage of our framework, highlighted in this comparison, is that it does not rely on pre-specified constant parameters, allowing the model to better adapt to evolving pandemic conditions.

## 1. INTRODUCTION

Case data for infectious diseases often misrepresent the true infectious population, both in terms of total burden and demographic distribution. The true number of infections is likely much higher than confirmed case counts due to under-ascertainment of mild or asymptomatic cases, limited testing availability, policy changes (regarding testing or otherwise), and data entry challenges within healthcare systems. For instance, an estimated 25% of SARS-CoV-2 infections worldwide were asymptomatic or mildly symptomatic (Alene et al. 2021) and therefore went unreported. We use the term *under-reporting* to describe any discrepancy between total infections and those captured in the data. This encompasses both under-ascertainment (eg infected individuals not seeking medical care) and under-reporting (eg cases not being entered into the notification system, incorrect diagnoses) (Bracher and Held 2021). Beyond these challenges, testing data may under-represent individuals in a community due to factors such as limited and inequitable access to testing, as well as sociodemographic biases related to sex, age, and education (Pongou et al. 2022).

These issues inherent in case data complicate efforts to estimate critical infectious disease metrics such as reproduction rates, reporting probabilities, vaccine efficacy, disease incidence, and prevalence. Accurate estimation of these quantities is essential for public health authorities to implement effective mitigation policies, protect and inform the public, and assess a pandemic’s impact, both retrospectively and in real-time. Inference on these quantities requires extending relevant infectious disease transmission frameworks to account for under-reporting and other complexities of disease dynamics. This often involves integrating auxiliary data sources—such as serological surveys and wastewater surveillance data—for model validation, calibration, or identifiability (Quick et al. 2021; Watson et al. 2024). In this paper, we focus on building a flexible framework for estimating infected cases (ie epidemic curve reconstruction), as well as time-varying case reporting probabilities and reproduction rates.

A simple and effective model for infectious disease transmission is the Poisson autoregressive (PAR) model of Held et al. (2005). These models have gained recent popularity due to their theoretical connection with discrete-time Susceptible-Infectious-Removed (SIR) compartmental models, which are widely used in epidemiology (Bauer and Wakefield 2018), as well as advancements in their multivariate extensions (Armillotta and Fokianos 2024). In their simplest form, PAR models assume that case numbers follow a conditional Poisson distribution, with the expected value determined by the sum of unexplained cases and a positive multiple of past cases. This model has been applied to various infectious diseases, including influenza, measles, and Covid-19 (Dunbar and Bekker-Nielsen Held 2020). Additionally, PAR frameworks are flexible and can easily be extended to include multiple time lags of past cases, spatially correlated random effects, covariates, and other important model structures (Bracher and Held 2022; Slater et al. 2025). Although we will not use them here, compartmental models are also commonly used for infectious disease modeling, relying on either ordinary differential equations or stochastic processes.

Because of under-reporting, fitting a PAR model directly to confirmed case counts can result in biased parameter estimates or a poor representation of true infection dynamics (Slater and Bebeziqi 2025). A widely adopted alternative model treats reported case counts as a Binomial sampling process, where the true infections and the reporting probabilities define the Binomial distribution (Bracher and Held 2021; Quick et al. 2021). In this hierarchical framework, the PAR model is applied to the latent, integer-valued infection counts. We refer to this extension as the *PAR model with binomial thinning* or the *under-reported PAR model*. A key challenge in applying such models is that reported case data typically provide limited information about the true number of infections and underlying disease dynamics, resulting in considerable uncertainty in epidemic reconstructions (Mohring et al. 2024). Moreover, reported cases tend to represent individuals more likely to be tested. Statistical inference in these models can be significantly improved by incorporating auxiliary data sources that better reflect the infectious population, such as wastewater and serological survey data.

Jointly modeling under-reported confirmed case counts and auxiliary data for epidemic curve reconstruction has been studied within the PAR framework. An expectation-maximization (EM) algorithm using seroprevalence data to calibrate the conditional expectation of infections in the E-step of the algorithm was developed by Quick et al. (2021). This framework is tailored for *daily* infection case counts and accounts for delays between infection, onset, and reporting, while incorporating multiple serological surveys to estimate time-varying prevalence, reporting probability, incidence, and the reproduction number. In Watson et al. (2024), a joint model for under-reported daily case counts and wastewater data was developed to estimate the reproduction rates, incidence, and reporting probabilities. The marginal posterior of the model’s hidden states (eg reporting probabilities, reproduction rates, and number of infections) given the data was estimated using a fixed-lag bootstrap filter algorithm, implemented within the particle marginal Metropolis-Hastings Markov Chain Monte Carlo (MCMC) algorithm to sample the joint posterior of the full model parameters. A limitation of this model is the requirement to preset parameters, including a fixed parameter relating wastewater to the number of infections, with results shown to be sensitive to its choice.

Additionally, the stochastic susceptible-exposed-infectious-recovered (SEIR) framework has been extended in Miyazawa et al. (2024) and Proverbio et al. (2022) to account for both under-reported infection counts and wastewater observations. These models are calibrated using an extended Kalman filter and provide estimates for key metrics such as reproduction numbers, reporting probabilities, and infected case numbers. In general, these models incorporate additional compartments to introduce auxiliary data, however they often make restrictive assumptions, rely on several pre-specified parameters, and are challenging to adapt to flexible spatiotemporal frameworks or models with covariates (Quick et al. 2021).

We propose a hierarchical Bayesian framework for epidemic curve reconstruction using under-reported confirmed case counts and wastewater data. This framework extends the under-reported PAR model by representing the reproduction rate as the multiplicative change in the mean virus concentration in wastewater, modeled within the wastewater framework introduced by Somerset and Brown (2024). Reported case data are influenced by testing behavior and other unmeasured biases. If this complex reporting process is misspecified, fitting a fully joint model risks contaminating inference on wastewater parameters, which are more likely to reflect the population of interest. To address this, we propose an epidemic curve reconstruction framework that fits the wastewater model separately and incorporates its posterior samples as data in the binomially thinned PAR model. This approach, known as modular Bayesian inference, allows wastewater data to inform parameters in the infectious disease model while preventing feedback from confirmed case counts to the wastewater model (Plummer 2015).

To facilitate Bayesian computation, we approximate discrete distributions in the binomially thinned PAR model using a normal-normal approximation (Slater et al. 2025), enabling the use of methods such as Hamiltonian Monte Carlo (HMC) and Adaptive Gauss-Hermite Quadrature (AGHQ). We validate our models through out-of-sample forecasts and, where available, by comparing results to serosurvey-based cumulative incidence estimates from Slater et al. (2024) and epidemic curve reconstructions from Watson et al. (2024).

## 2. METHODS

### 2.1. Under-reported Poisson autoregressive model for case counts

We denote $ Y_{j} $ and $ I_{j} $ as the total number of confirmed and the true number of infections in week $ j $, respectively. We model the confirmed case counts as a binomially thinned process, with new infections following a PAR model. The hierarchical model for confirmed case counts is


(2.1) \begin{align*}\begin{aligned} Y_j\mid I_j, \pi_j\sim &\mathrm{Binomial}(I_{j},\pi_j)\\I_{j}\mid I_{k}; k < j\sim &\mathrm{Poisson}(R_j I_{j-1})\\ {\rm logit} (\pi_j)\sim &\mathrm{RW}_1(\sigma_\pi)\\R_j = &\tilde\mu_j/\tilde\mu_{j-1}.\end{aligned}\end{align*}

In (2.1), $ 0 < \pi_{j}\leq 1 $ is the reporting fraction for week $ j $. On the logit scale, it evolves discretely over time as a non-stationary, first-order Gaussian random walk with standard deviation $ \sigma_{\pi} $, allowing for sustained changes in reporting behavior rather than mean-reverting fluctuations. Conditioned on the past number of infections, the number of new infections in week $ j $ depends on the number of infections the previous week $ I_{j-1} $ and the reproduction rate (or number of secondary infections) $ R_{j} $. Epidemic models of this sort often include a “spark” term allowing for spontaneous infections, which we have excluded as we are considering only the time period where the epidemic is established and we are considering fairly large populations where $ I_{j} $ will be large.

The key element of the joint case count and wastewater model is that changes in RNA levels in wastewater reflect changes in the number of people infected in the community. As more people are infected, more viral RNA is shed and concentrations rise; when fewer are infected, concentrations fall. To capture this relationship, we link the reproduction rate to the multiplicative change in the underlying viral load measured in wastewater, $ R_{j}=\tilde{\mu}_{j}/\tilde{\mu}_{j-1} $. Section 2.3 builds on this idea by moving from a continuous-time description to this stepwise ratio. However, first we describe the wastewater model in Section 2.2 from which $ \tilde{\mu}_{j} $ are inferred from noisy wastewater measurements.

### 2.2. Wastewater model

The model used for wastewater concentrations is that developed by Somerset and Brown (2024). Let $ G_{it} $ denote the viral concentration, measured as the number of gene copies in either wastewater liquid or solids, at monitoring station $ i $ on day $ t $. The full model is


(2.2) \begin{align*}\begin{aligned} G_{it} &\sim\mathrm{Gamma}\left(\kappa^{-2},\ \eta_{it}\kappa^2\right)\\\eta_{it} &=\mu_{i}(t)\cdot\exp(Z_t)\\\log[\mu_{i}(t)] &=\beta_i + U_{i}(t) + V(t)\\\mathrm{Cov}[U_i(t+h), U_i(t)] &=\sigma_u^2\mathcal{M}(h;\phi, 1.5)\\ V(t) &\sim\mathrm{IWP}_3(\sigma_v)\\ Z_t &\sim\mathrm{Normal}(0, \sigma_z^2).\end{aligned}\end{align*}

Each station has a station-specific mean concentration $ \eta_{it} $ which depends on a common trend $ V(t) $ and station-level deviations from the trend $ U_{i}(t) $. In (2.2), $ \beta_{i} $ denotes the fixed effect for station $ i $, parameterized so that $ V(0) $ is the average station effect. The common trend $ V(t) $ is modeled as a non-stationary, third-order Integrated Wiener Process (IWP) with variance $ \sigma_{v}^{2} $, a smooth Gaussian process which is akin to using an integrated squared third-derivative penalty in a penalized likelihood (see Zhang et al. 2024). Somerset and Brown (2024) chose a non-stationary process to allow flexibility in capturing the global wastewater signal during the pandemic, which exhibited multiple large-scale waves. The third-order IWP also produces a quadratic trend in the smoothing limit ($ \sigma_{v}\to 0 $), aligning with our prior belief that each COVID-19 wave is reasonably quadratic on the log scale, with new waves arising when additional episodes occur. Station-specific effects are modeled using independent zero-mean stationary Gaussian processes $ \{U_{i}(t),t\geq 0\} $, all sharing a common Matérn covariance with shape parameter 1.5, variance $ \sigma^{2}_{u} > 0 $, and scale $ \phi_{u} > 0 $. These viral signals are modeled as stationary processes because they describe deviations from the global trend with a tendency to return to the mean level. Somerset and Brown (2024) chose the Matérn(1.5) covariance because of it being a simple one-parameter stationary process with a continuous first derivative. Further details on the implementation of (2.2) regarding $ U_{i}(t) $ and $ V(t) $ are provided in Somerset and Brown (2024). The term $ Z_{t} $ is an time-independent random effect which is common to all monitoring stations, reflecting daily variation in environmental factors such as precipitation. Additional station-level variation is reflected in the Gamma distribution of the observations, with $ \eta_{it} $ being the expected value and $ \kappa={\rm sd}(G_{it})/{\rm E}(G_{it}) $ the coefficient of variation (or equivalently $ \kappa^{-2} $ is the Gamma shape parameter).

An aggregate, population-weighted wastewater signal for the study area, $ \bar{\mu}(t) $, used to compute the $ \tilde{\mu}_{j} $ in (2.1), is given by


(2.3) \begin{align*}\bar{\mu}(t)=\sum\limits_{i}P_{i}\mu_{i}(t)\end{align*}

where $ P_{i} $ is a weight proportional to the population of $ i $-th wastewater station’s catchment area. An alternative definition of the wastewater signal considers only the common trend with


(2.4) \begin{align*}\bar{V}(t)=\exp[V(t)]\cdot\sum\limits_{i}P_{i}e^{\beta_{i}}.\end{align*}

This second definition is equivalent to omitting the station-level effects $ U_{i}(t) $ from $ \bar{\mu}(t) $. The term involving $ \beta_{i} $ is, in effect, a normalizing constant which does not change over time. The first definition, $ \bar{\mu}(t) $, would be expected to have greater estimation uncertainty, particularly when the number of stations is small and the parameters $ \sigma_{u} $ and $ \phi_{u} $ are not well identified.

### 2.3. Wastewater-based reproduction rates

The assumption in (2.1) is motivated by the belief that changes in RNA levels in wastewater, $ \bar{\mu}(t) $, should be governed by a continuous-time reproduction rate, $ R(t) $:


(2.5) \begin{align*}\bar{\mu}(t+h)\approx R(t)^{h}\bar{\mu}(t).\end{align*}

When $ R(t) > 1 $, the viral signal is expected to increase over time, with growth compounding as $ h $ increases; for example, $ R(t)=2 $ indicates that the signal would double each time unit. Conversely, when $ R(t) < 1 $, the signal is expected to decline, with $ R(t)=0.5 $ indicating a halving each time unit. Taking the limit as $ h\to 0 $ in (2.5) yields a continuous-time measure for the reproduction rate:


(2.6) \begin{align*} R(t)=\lim_{h\to 0}\left(\frac{\bar{\mu}(t+h)}{\bar{\mu}(t)}\right)^{1/h}=\exp\left(\frac{\bar{\mu}^{\prime}(t)}{\bar{\mu}(t)}\right).\end{align*}

Here, $ \bar{\mu}^{\prime}(t) $ is the instantaneous rate of change of the wastewater signal. Dividing by $ \bar{\mu}(t) $ expresses this change relative to the current size, which is equivalent to the logarithmic derivative of $ \bar{\mu}(t) $. This relative measure is positive when the signal is increasing, negative when it is decreasing, and captures the speed of exponential change regardless of the absolute magnitude of the wastewater signal.

The station-specific wastewater signals $ \mu_{i}(t) $ are modeled using a twice-differentiable, non-stationary common trend $ V(t) $, along with once-differentiable station-specific deviations $ U_{i}(t) $, allowing for inference on $ \bar{\mu}^{\prime}(t) $. On the discrete scale, as used in (2.1), the weekly reproduction rate is defined as:


(2.7) \begin{align*}\tilde{R}_{j}=\frac{\int_{W_{j}}\bar{\mu}(t)\, dt}{\int_{W_{j-1}}\bar{\mu}(t)\, dt}=\frac{\tilde{\mu}_{j}}{\tilde{\mu}_{j-1}}.\end{align*}

Here, weekly aggregation is required because reported case counts are only available at the weekly scale, whereas wastewater data are observed daily. In (2.7), $ W_{j} $ denotes the week-long time interval for the $ j $th observation. This discrete form follows from (2.5) under the assumption that $ R(t) $ remains approximately constant over each weekly interval. Both (2.6) and (2.7) provide direct methods for inferring shifts in disease transmission dynamics by tracking changes in the wastewater signal.

### 2.4. Modular Bayesian inference for epidemic curve reconstruction

Bayesian inference in our setting involves sampling jointly from the posterior distribution of all model parameters. This posterior can be written as:


(2.8) \begin{align*} p(\pi_{j},I_{j},\kappa, \eta_{it},\bar{\mu}(t)|G_{it},Y_{j})=\underbrace{p(\pi_ {j},I_{j}|Y_{j},\bar{\mu}(t))}_{\text{Infection model}}\times\underbrace{p(\bar{\mu}(t),\kappa, \eta_{it}|G_{it},Y_{j})}_{\text{Wastewater model}},\end{align*}

such that the first term corresponds to the infection model and the second to the wastewater model. This factorization makes explicit that parameters of the wastewater component are conditionally dependent on reported case counts through $ Y_{j} $. While a fully joint specification has the advantage of propagating information across modules, it also creates the possibility that misspecification in one component, such as the infection model, influence estimates in the other (Jacob et al. 2017).

We view wastewater data as providing a more representative picture of community infections than reported case data, which is affected by changes in testing behavior, data entry errors, and systematic differences between individuals who do and do not get tested. Because this complex reporting process is not modeled explicitly, the reported case series cannot be relied upon to estimate the autoregressive parameter $ R_{j} $ of the latent infection process, which characterizes transmission dynamics in the entire infectious population. We therefore rely on the wastewater model’s estimate of $ R_{j} $, and seek to prevent contamination by removing its dependence on $ Y_{j} $.

Cut models (or modular inference) are an alternative to full Bayesian probability models, modulating the flow of information from data to parameters (Plummer 2015; Jacob et al. 2017). To achieve this, we first fit the wastewater model (2.2) separately to obtain posterior samples $ \bar{\mu}(t)^{(s)} $ for $ s\,=\,1, \ldots, S $ from $ p(\bar{\mu}(t),\kappa, \eta_{it}|G_{it}) $. Then, for each $ s\,=\,1, \ldots, S $, we sample from $ p(\pi_{j},I_{j}|Y_{j},\bar{\mu}(t)) $, treating $ \bar{\mu}(t)^{(s)} $ as $ \bar{\mu}(t) $ when fitting the infection model (2.1). The combined samples over $ s\,=\,1, \ldots, S $ are samples from the cut posterior, which is equivalent to (2.8) but with the dependence on $ Y_{j} $ removed from the wastewater model (Plummer 2015).

### 2.5. Implementation and inference

Inference for the wastewater model in (2.2) and the infection count model in (2.1) is conducted within an approximate Bayesian framework using the AGHQ algorithm introduced by Stringer et al. (2023). This method applies a nested combination of Gaussian, Laplace, and adaptive quadrature approximations, offering greater computational efficiency and better scalability with larger datasets compared to other approximation techniques (Stringer et al. 2023). Models are implemented using C++ templates, with automatic differentiation used to compute the derivatives required for Laplace approximations.

Computations for the wastewater models in Sections 3.1 and 3.2 were done using 3 quadrature points and 500 posterior samples. Modular inference for the infection count models were conducted with 10 quadrature points and 250,000 samples from the cut posterior. The integrals in (2.7) were approximated using sums over a daily time grid. The integer-valued parameters in these under-reported PAR models introduces challenges in model fitting. State-of-the-art inference methods, such as Bayesian HMC, INLA, AGHQ, and Kalman filters, operate under continuous latent variables. A moment-matching approach for fitting the simplest form of the PAR model, in the presence of under-detection, was proposed by (Bracher and Held 2021). However, this method does not directly accommodate increased model complexities, such as the logistic random walk model on the reporting probabilities. To enable inference on latent discrete parameters in (2.1) using the AGHQ algorithm, a normal-normal approximation was applied to the Binomial and Poisson distributions, resulting in the conditional distributions $ Y_{j}|I_{j},\pi_{j}\sim{\rm Normal}(\pi_{j}I_{j},\pi_{j}(1-\pi_{j})I_{j}) $ and $ I_{j}|R_{j},I_{j-1}\sim{\rm Normal}(R_{j}I_{j-1},R_{j}I_{j-1}) $ (Slater and Bebeziqi 2025). This approximation becomes more accurate with larger case counts $ Y_{j} $. All statistical analyses were carried out using R version 4.4.2, with the packages OSplines version 0.1.1, aghq version 0.4.1, and TMB version 1.9.16.

### 2.6. Prior distributions

Standard deviation parameters for the wastewater model are all given exponential priors, motivated by the penalized complexity argument of Simpson et al. (2017). The standard deviation terms $ \sigma_{u} $, $ \sigma_{z} $, and the coefficient of variation $ \kappa $ are Exponential with median 0.5, as their associated random effects operate on the log scale the prior is only weakly informative. The Integrated Wiener Process for the common trend $ V(t) $ has a variance parametrized by the $ h $-step ahead prediction variance $ \sigma_{v}(h)={\rm var}[V(t\,+\,h)|V(u),u\,< \,t] $ from Zhang et al. (2024), with the 20-d predictive standard deviation $ \sigma_{v}({\text{20 d}}) $ being Exponential with median 2. We assign an Exponential prior to the range parameter $ \phi_{u} $ of $ U_{i}(t) $ with a median of 50 d. These priors reflect our belief that variation in Covid-19 shedding is primarily due to region-wide changes in viral load encompassed in $ V(t) $ and less affected by station-level factors, independent day-to-day variation, or observation error. The boundary conditions for $ V(t) $ at $ t_{0} $ are specified by normal prior distributions. Specifically, $ V(t_{0}) $, $ V^{\prime}(t_{0}) $, and $ V^{\prime\prime}(t_{0}) $, along with the station-specific fixed effects $ \beta_{i} $, follow $ {\rm N}(0,10^{2}) $, representing a non-informative prior for viral concentrations.

In the under-reported PAR model, we specify weakly informative priors for $ \sigma_{\pi} $, the initial infection count $ I_{0} $, and the initial reporting probability $ \pi_{0} $. The standard deviation $ \sigma_{\pi} $ of the random walk on the log odds of reporting follows an Exponential prior with a median of 1, reflecting the belief that the reporting probability is unlikely to change significantly from week to week. The initial reporting probability, $ \pi_{0} $ has a logit-Normal prior distribution $ {\rm logit}(\pi_{0})\sim{\rm Normal}(0,1) $. The prior density of $ \pi_{0} $ is concentrated around 0.5, with 95% quantiles at 0.12 and 0.88. We assign a Normal prior on $ I_{0} $ with mean equal to twice the confirmed case count at time 0 and standard deviation of 500. For example, the confirmed case count in Toronto for the week ending October 10, 2020, was 1,762, implying that the 95% quantiles for the prior of $ I_{0} $ are approximately 2,544 and 4,503.

## 3. APPLICATIONS

### 3.1. Toronto

**Fig. 1.**
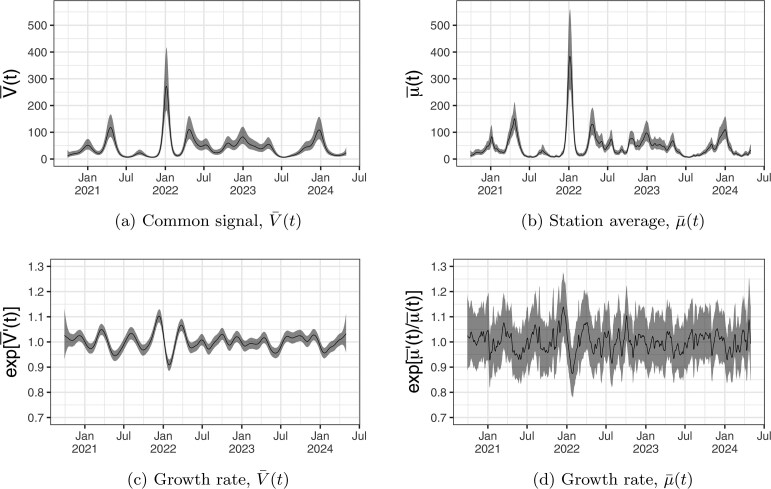
Signals and growth rates inferred from Covid-19 concentrations in wastewater across Toronto from October 2020 to April 2024. Shown are the 95% credible intervals (grey) and posterior medians (black) for the scaled common trend $ \bar{V}(t) $ (left panels) and population-weighted station average $ \bar{\mu}(t) $ (right panels).

Figure 1 shows results from applying the wastewater model from Section 2.2 to wastewater monitoring data from Toronto, Canada, from September 28, 2020, to April 30, 2024. Data are reported by the Public Health Agency of Canada (Public Health Agency of Canada 2024), and represent the daily average of two technical replicates measuring SARS-CoV-2 RNA gene-target N2 concentrations (in gc/mL) at four sampling sites across Toronto. Sampling times are irregular, with a median frequency of two samples per week per site. Across the 1,311-d study period, samples were collected on 430 d, with most non-sampled days falling on Fridays and Saturdays.

**Fig. B1.**
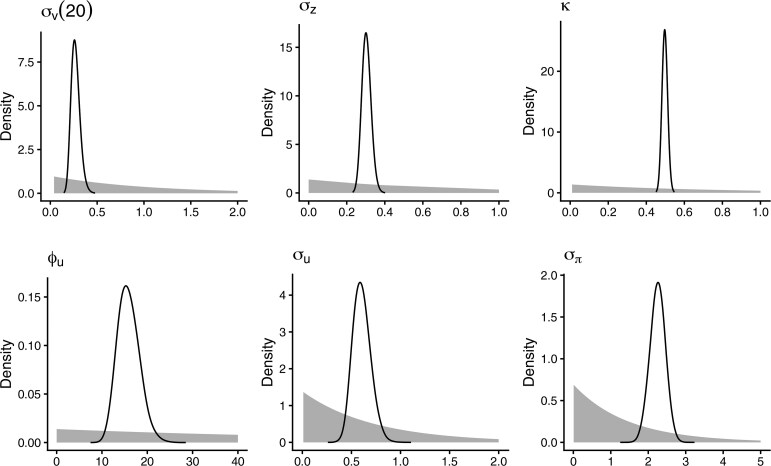
Priors and posteriors distributions for hyperparameters from the wastewater model and under-reported PAR model for Covid-19 in Toronto. Posterior distributions are shown in black and the respective prior distributions are shaded in grey.

Posterior prediction intervals for the two specifications of Toronto’s Covid-19 signal, $ \bar{\mu}(t) $ in Fig. 1a and $ \bar{V}(t) $ in Fig. 1b, are shown along with their growth rates, $ {\rm exp}[\bar{\mu}^{\prime}(t)/\bar{\mu}(t)] $ in Fig. 1c and $ {\rm exp}[\bar{V}^{\prime}(t)] $ in Fig. 1d. The population-weighted signal $ \bar{\mu}(t) $ includes the station-specific terms $ U_{i}(t) $, which results in a rougher signal and more uncertainty in the growth rate than $ \bar{V}(t) $. The most prominent feature is the sharp rise in the signals during first Omicron wave in late 2021 and early 2022. The growth rates both show a clear positive effect in late 2021 and turn negative during the first weeks of 2022, albeit with considerable uncertainty for $ \bar{\mu}(t) $. Posterior and prior distributions for the wastewater model parameters are shown in Fig. B1. The posterior density for the Matérn range parameter is centered at approximately 16 d, suggesting that autocorrelation in $ U_{i}(t) $ becomes negligible after about half a month.

**Fig. 2.**
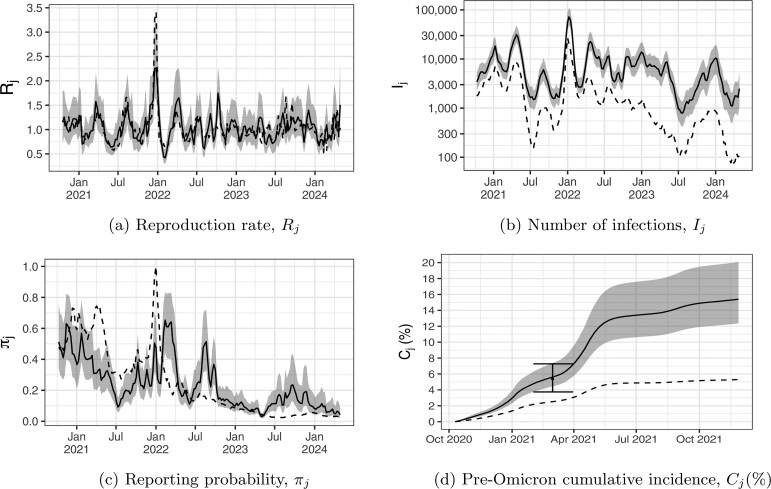
Results for Covid-19 in Toronto, with posterior medians (solid lines) and 95% credible intervals (grey regions). Panel a) shows reproduction rates $ R_{j} $ (solid line) and a crude estimate based on the lagged ratios of confirmed case counts (dashed line). Panels b), c), and d) show inferred new infections, probabilities, and cumulative incidence, respectively. Confirmed case counts and cumulative confirmed case counts are shown as dashed lines in b) and d). Weekly laboratory testing numbers, scaled to their maximum value over the observation period, are shown as a dashed line in panel c). The error bar in panel d) is seroprevalence.

**Fig. B2.**
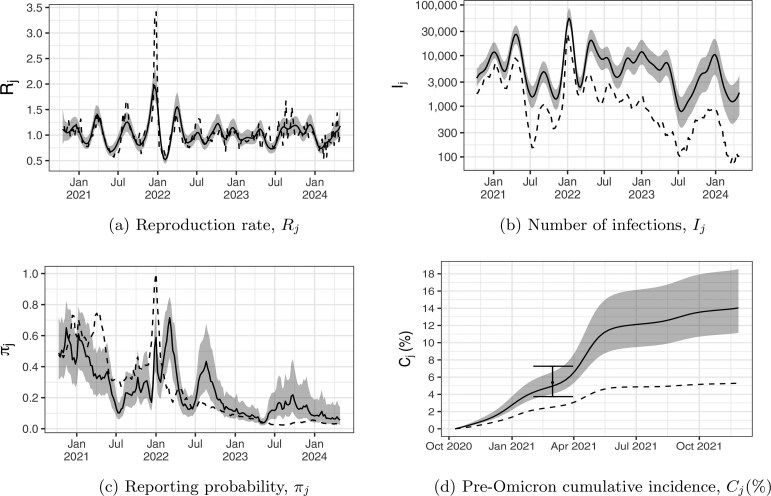
Results for Covid-19 in Toronto, with posterior medians (solid lines) and 95% credible intervals (grey regions). The defined station-specific signal is based on the common signal and station intercepts. Panel a) shows reproduction rates $ R_{j} $ (solid line) and a crude estimate based on the lagged ratios of confirmed case counts (dashed line). Panels b), c), and d) show inferred new infections, probabilities, and cumulative incidence, respectively. Confirmed case counts and cumulative confirmed case counts are shown as dashed lines in panels b) and d). Weekly laboratory testing numbers, scaled to their maximum value over the observation period, are shown as a dashed line in panel c). The error bar in panel d) is seroprevalence.

Figure 2 reconstructs Toronto’s Covid-19 epidemic curve, combining laboratory-confirmed case counts with the wastewater model results. Here, we use the station-average wastewater signal as defined in (2.3), with results based on the common trend from (2.4) shown in Fig. B2. The four sub-figures of Fig. 2 have the posterior medians and 95% credible intervals as solid lines and grey shaded regions, respectively. Reproduction rates, $ R_{j} $, are shown in Fig. 2a, with the dashed line being the ratio of observed case counts $ Y_{j}/Y_{j-1} $ (a simple estimate of $ R_{j} $). In Fig. 2b, the solid line represents weekly new infections $ I_{j} $, while the dashed line represents confirmed case counts. Figure 2c shows posterior summaries of the reporting probability $ \pi_{j} $, with weekly COVID-19 testing counts scaled to their maximum value of 26,855 over the observation period. These scaled testing numbers are shown as a dashed line, with the peak occurring near the end of December 2021. The testing numbers reflect changes in testing behavior over time; scaling them to lie between 0 and 1 allows them to be plotted on the same axis as the model-based $ \pi_{j} $, enabling visual comparison of temporal patterns rather than direct comparison of magnitudes. The median of $ \pi_{j} $ roughly follows the trend of the scaled test counts, decreasing in July 2021 and continuing to decline after the emergence of Omicron.

The most notable discrepancy between the model predictions and their associated crude estimates occurs at the start of the Omicron wave in December 2021. In Fig. 2a the peak of the raw ratios $ Y_{j}/Y_{j-1} $ is over 50% higher than the modeled estimate for $ R_{j} $. Weekly variation in numbers of individuals tested could explain some of this difference. Figure 2c shows a sharp and brief spike in the reporting probability $ \pi_{j} $ at the same time the weekly number of Covid-19 tests (dashed line) climbs. Partly due to rising public concern over the new variant, the number of tests performed rose to 88,502 for the week ending 25 December 2021, a increasing 28% from 68,887 tests the previous week. The ratio $ Y_{j}/Y_{j-1} $ that week is $ 15,321/4,487\approx 3.4 $, which overestimates the increase in infections as the denominator is a smaller proportion of total infections than the numerator. The joint wastewater model predicts $ R_{j} $ for the same week to be 1.86 (95% credible intervals 1.60-2.21), a level which is lower but still alarming.

To validate the reconstructed epidemic curves we compare the cumulative number of infections $ C_{j}=\sum_{k\leq j}I_{k} $ in Fig. 2d to antibody prevalence estimated from serology data. The sideways H-shaped error bar shown in March 2021 on Fig. 2d gives a 95% confidence interval for Ontario’s seroprevalence from Slater et al. (2024). Our analysis begins in October 2020 when wastewater data first became available, and the cumulative incidence excludes cases from the initial wave from March to October 2020. There were 150,000 positive tests reported prior to October 2020, as this is a small fraction of the 2 million positive tests at the end of December 2021. The width of the error bars in Fig. 2d were extended to span February to March in order to illustrate the temporal uncertainty associated with the seroprevalence estimate. In the seroprevalence study, 75% of samples were returned by February 9, 2021, and 25% by the end of January 2021, supporting this date range (Brown et al. 2024). Our model estimates a cumulative incidence of 5.56% [4.39%–7.29%] by February 27, 5.75% [4.54%–7.53%] by March 6, and 6.85% [5.42%–8.96%] by March 27, 2021. The median and 95% credible interval from seroprevalence data, 5.38% [3.74%–7.27%], align well with our early March, late February estimates. Given Toronto’s greater population density and mobility patterns, its cumulative incidence is likely slightly higher than Ontario’s, which is reflected in our results.

**Fig. 3.**
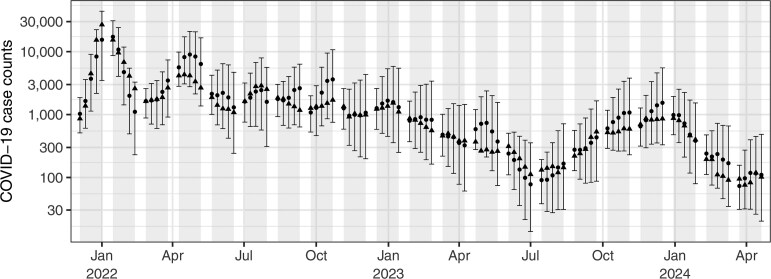
Posterior medians and 95% credible intervals for forecasted reported COVID-19 case counts in Toronto. Shaded regions indicate 5-wk forecast periods, and triangles represent the recorded, held-out case counts.

We further validate our framework through near-term forecasts of reported Covid-19 case counts. Using the wastewater-based $ R_{j} $ estimates from the wastewater model, we train the epidemic model and forecast reporting probabilities up to 5 wks ahead, following its random walk structure. These forecasted probabilities, together with posterior (non-forecasted) $ R_{j} $ values, are then used to simulate reported case counts over this held-out, 5-wk prediction period. Figure 3 shows the median predicted case counts (circles) and their 95% credible intervals (error bars) on a decimal logarithmic scale. This validation exercise was repeated 21 times across the entire time series, with shaded regions indicating each 5-wk held-out forecasting period. In this figure, the true reported case counts (triangles) fall within the 95% credible intervals, often aligning closely with the median posterior predictions.

### 3.2. New Zealand

In this section, we estimate New Zealand’s Covid-19 epidemic curve and compare results to those from Watson et al. (2024). The comparison model in Watson et al. (2024) jointly estimates daily wastewater data and case counts within the PAR framework, modeling under-reported case counts using a negative binomial distribution. The expectation of this distribution is expressed as a linear combination of past infections, determined by a known infection-to-reporting delay distribution. The number of current infections, $ I_{t} $, is a latent variable modeled as a Poisson random variable with mean $ R_{t}\sum_{u\,=\,1}^{t-1}g_{u}I_{t-u} $, where $ g_{u} $ is a pre-specified generation time distribution and $ R_{t} $ denotes the instantaneous reproduction rate. The prior distribution for $ R_{t} $ is given by $ N_{(0, \infty]}(R_{t-1},\sigma_{R}R_{t-1}) $. Wastewater data, $ W_{t} $, is modeled using a Gamma distribution, with an expectation proportional to a known linear function of past infections up to time $ t $. Their model parameter $ \alpha $, shown in subsequent plots, is the user-specified proportionality constant and represents the average total genome copies shed per infection.

**Fig. 4.**
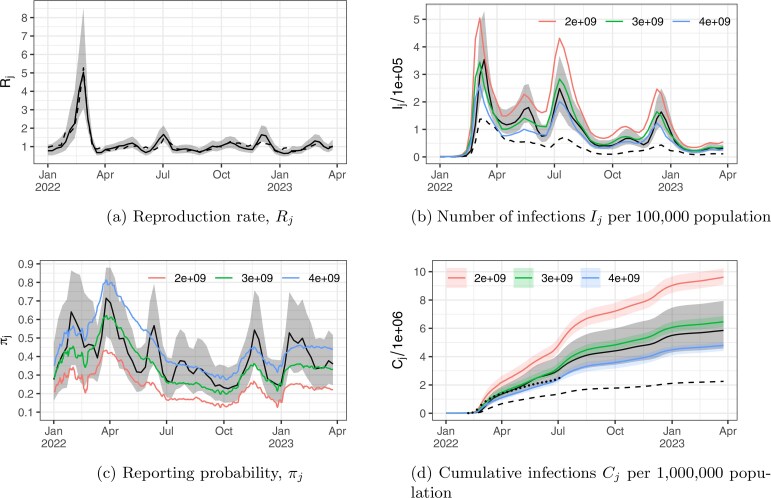
Wastewater model and epidemic curve reconstruction results for Covid-19 in New Zealand. Panel a) shows the posterior medians for wastewater-based reproduction rates (solid line), with 95% credible intervals shaded in gray, and a crude estimate based on lagged confirmed case counts (dashed line). Panels b), c), and d) display the posterior medians from the epidemic model for new infections, reporting probabilities, and cumulative infections, respectively, with 95% credible intervals shaded in gray. Confirmed case counts and cumulative confirmed case counts are plotted as dashed lines in b) and d). Results from the comparison model are displayed in panels b), c) and d), color-coded by their model parameter $ \alpha $, with corresponding 95% credible intervals shaded in the same colors in panel d). Black dots in panel d) represent the cumulative per capita case counts for a cohort of routinely tested border workers, scaled to population size.

**Fig. B3.**
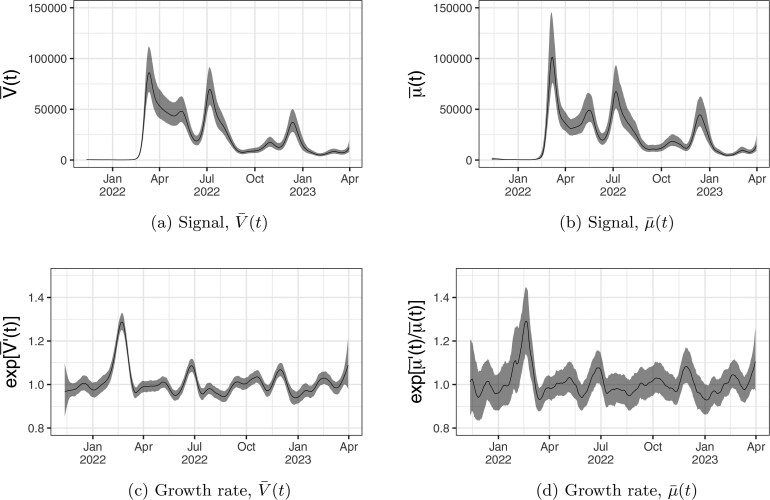
Signals and growth rates inferred from Covid-19 concentrations in wastewater across 134 wastewater sites in New Zealand from November 12, 2021, to March 31, 2023. Shown are the 95% credible intervals (grey) and posterior medians (black) for the scaled common trend $ \bar{V}(t) $ (left panels) and population-weighted station average $ \bar{\mu}(t) $ (right panels).

Results from the wastewater model are shown in Fig. B3 and discussed in Appendix Section A. The medians of the posterior distributions for the wastewater-based reproduction rates, $ R_{j} $, are shown as a solid line in Fig. 4a, with 95% credible intervals shaded in gray. The dashed line represents the simple estimate of $ R_{j} $, calculated as the ratio of $ Y_{j} $ to $ Y_{j-1} $. The wastewater-based reproduction rate peaks during the first Omicron wave in the week ending February 26, 2022, with a median estimate of 5.04 (95% credible interval: [3.07, 8.53]). The simple estimate of $ R_{j} $ also reaches its peak at this time, at 5.27. Overall, the median posterior estimate and the crude estimate track each other closely.

Figure 4 summarizes the posterior distributions for New Zealand’s Covid-19 epidemic metrics. In Fig. 4b, the median and 95% credible intervals for $ I_{j} $, scaled by a factor of $ 10^{5} $, are shown in black and shaded in gray, respectively. The estimates from the comparison model for three different values of $ \alpha $ are displayed in red, green, and blue, with higher values of $ \alpha $ producing curves with smaller amplitudes. Both models produce Covid-19 epidemic curves characterized by three distinct waves in 2022, occurring in March, July, and December. The peaks of the first and third Omicron waves are misaligned between the two models, while the second wave shows alignment. Observed weekly case counts are shown as a black dashed line. For the first and third waves, all three estimates from the comparison model at their peaks fall within our model’s 95% credible interval at the corresponding peak times, indicating agreement on wave amplitude. For the second Omicron wave, the mean estimates from the comparison model at $ \alpha\,=\,3\times 10^{9} $ and $ \alpha\,=\,4\times 10^{9} $ lie within our model’s 95% credible interval.

Posterior summaries for the reporting probabilities from both models are shown in Fig. 4c, showing moderate agreement across models. In Fig. 4d, posterior medians and 95% credible intervals for cumulative $ I_{j} $ are presented for both models. The 95% credible intervals from our model, shaded in gray, capture the mean estimates from the comparison model at $ \alpha\,=\,3\times 10^{9} $ and $ \alpha\,=\,4\times 10^{9} $, and falls below the estimates at $ \alpha\,=\,2\times 10^{9} $ after July, 2022. The dashed line represents the cumulative number of infections computed from observed case counts, while the black dotted line shows the number of cases per capita among regularly tested border workers, scaled to New Zealand’s population size, as used in Watson et al. (2024) for model validation. Although the width of our credible interval for the cumulative number of cases is larger than that of the comparison method for a given $ \alpha $, the uncertainty in $ \alpha $ itself is not accounted for. Consequently, the cumulative number of cases estimated from border workers does not appear to agree with the comparison method for any single value of $ \alpha $ over the observation period. The value of $ \alpha $ that best fits the data seems to increase over time, which is evident from the fact that, for $ \alpha\,=\,2\times 10^{9} $, only 3 observations (13.6%) at the start of the epidemic fall within the 95% credible interval; for $ \alpha\,=\,3\times 10^{9} $, 12 observations (54%) are captured, with most missed during the second Omicron wave; and for $ \alpha\,=\,4\times 10^{9} $, 7 observations (31.8%) are captured, primarily at the start of the second wave. In contrast, our model captures 17 observations (77.3%), with 5 missed at the start of the epidemic, suggesting that the uncertainty in the cumulative number of cases is better represented.

## 4. DISCUSSION

In this paper, confirmed case counts are modeled within the PAR framework, with under-reporting accounted for through a Binomial thinning process. Relying solely on confirmed case data for epidemic curve reconstruction limits our ability to infer time-varying reporting probabilities and reproduction rates. Moreover, such models may introduce biases due to the under-representativeness of testing data. To address these limitations, an alternative approach is to integrate wastewater data into the framework, incorporating additional representative information on infectious disease dynamics into the model.

The statistical framework for viral concentrations in wastewater, introduced in Somerset and Brown (2024), enables inference on the underlying wastewater signal and its derivative. This signal is decomposed into a common trend and station-specific deviations, both modeled as differentiable Gaussian processes. In this paper, we define an approximation to the reproduction rate in the binomially thinned PAR model based on the logarithmic derivatives (relative growth rates) of the wastewater signals, which can be obtained from the aforementioned wastewater model. We demonstrate that these wastewater-based reproduction rates closely track simple estimates based on the ratio of lagged confirmed case counts. Deviations between these estimates are expected, and some discrepancies coincided with abrupt changes in testing frequency, as observed in Toronto.

We propose a statistical framework for epidemic curve reconstruction that involves fitting the wastewater model separately to obtain posterior samples of reproduction rates and incorporating these samples as data in the binomially thinned PAR model. This method, known as modular Bayesian inference, allows wastewater data to inform parameters in the infectious disease model while preventing feedback from confirmed case counts to the wastewater model. Given likely surveillance biases in case data, this strategy helps mitigate bias in the inference of wastewater parameters that could arise from the potential mis-specification of the case model.

The framework, used on Toronto’s confirmed case counts and wastewater data, showed good calibration with external estimates of cumulative incidence from seroprevalence data and out-of-sample predictions. We also applied our framework to New Zealand’s wastewater and confirmed case counts, using the same data as in Watson et al. (2024). Our results were generally consistent in estimating the magnitude of true infection counts and, consequently, the cumulative number of infections, further validating our framework. Moreover, the validation source used in that study, positive cases among regularly tested border workers, aligned more closely with our model.

A limitation of the comparison model is its reliance on the parameter $ \alpha $, which represents the average number of detectable genome copies shed by an infectious individual. This parameter must be pre-specified and is treated as a constant in their framework, with results shown to be sensitive to its choice. The authors note that varying $ \alpha $ provides plausible bounds on the total number of infections, which aligns with our findings, as our estimates fall within the bounds of their results. This highlights a key advantage of our framework: it does not require a fixed functional relationship between measured wastewater and infection counts. The average number of genome copies shed by infected individuals is unlikely to be constant, as it depends on factors such as variant type, vaccination status, and reinfection (Puhach et al. 2022, 2023). Instead, our model assumes that the growth rate of wastewater virus concentrations reflects the disease’s reproduction rate. This relationship remains robust to variations in the quantity of genetic material shed, provided that shedding patterns remain consistent across most of the population. This allows our model to better adapt to evolving pandemic conditions.

However, assuming consistent shedding patterns across a population also introduces a limitation in our framework, as viral shedding varies between individuals (Hoffmann and Alsing 2023). Moreover, shedding is influenced by factors such as vaccination status and the infecting viral variant. Individuals differ in their vaccination status, and different proportions of the population may be infected by distinct variants simultaneously. One possible alternative to our modeling assumption is to assume that, on average, reproduction rates can be approximated by wastewater-based rates. This can be incorporated into the framework by using the wastewater-based rate as the expectation of a normal prior for the reproduction rate.

The results presented in this paper focus on large cities or country-wide infection counts. When applying these models at the community level $ i $, the rate of infection at time $ j $, $ \lambda_{ij} $, is decomposed into additional components, including an exogenous component, a community-specific autoregressive term $ \lambda_{ij}^{\mathrm{ar}}=R_{ij}^{\mathrm{ar}}Y_{i, j-1} $, and a spatial component $ \lambda_{ij}^{\mathrm{spat}}=R_{ij}^{\mathrm{spat}}\sum_{k\neq i}w_{i, k}Y_{k, j-1} $ with spatial proximity weights $ w_{i, k} $ (Slater et al. 2025). In such cases, the relative growth rate of a community’s wastewater signal may not provide a reliable proxy for the disease’s reproduction rate. Instead, each reproduction rate in this decomposition is likely a weighted combination of community-specific wastewater signals, requiring the derivation of an appropriate mathematical relationship, which we leave for future work.

Another challenge at the community level arises from working with multivariate count time series that have a smaller number of cases. Inference for multivariate count time series is an active area of research. The computational methods used in this paper rely on a normal-normal approximation to the Binomial and Poisson distributions, as traditional Bayesian computation frameworks, such as HMC and INLA, do not support integer-valued parameters (eg the true number of infections). However, this approximation becomes less reliable when case counts are low, requiring alternative approaches, such as a customized sampler.

Finally, we reconstruct the epidemic curve on a weekly scale because case counts are typically reported as weekly totals. If daily reporting dates were available, the model could be extended to account for the day-of-the-week effect and for delays between infection and reporting (Quick et al. 2021; Watson et al. 2024). In our current formulation, these events are treated as occurring within the same week. Under the assumption that the serial interval (the time from symptom onset in a primary case to symptom onset in a person they infect) of COVID-19 is 1 wk, $ R_{j} $ can be interpreted as the time-varying effective reproduction number. At a daily resolution, however, the number of infections at time $ t $ would instead depend on earlier cases through a convolution with the serial interval distribution, which is treated as known in Quick et al. (2021).

## Supplementary Material

kxaf033_Supplementary_Data

## Figures and Tables

**Fig. B4.**
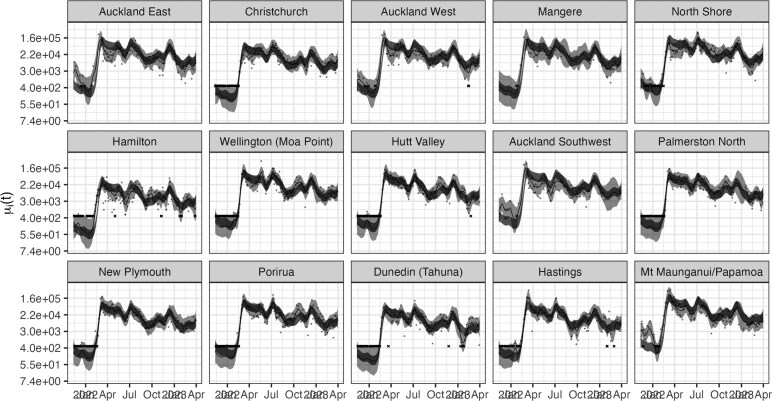
Posterior prediction intervals for Covid-19 signals at 15 wastewater sites in New Zealand. Each panel shows the posterior median of $ \mu_{i}(t) $ (black line) with its 95% credible interval (shaded light grey), observed data (black dots), censored data (black Xs), and the 95% credible interval for the scaled common trend $ \exp[\beta_{i}+V(t)] $ (shaded dark grey).

**Fig. B5.**
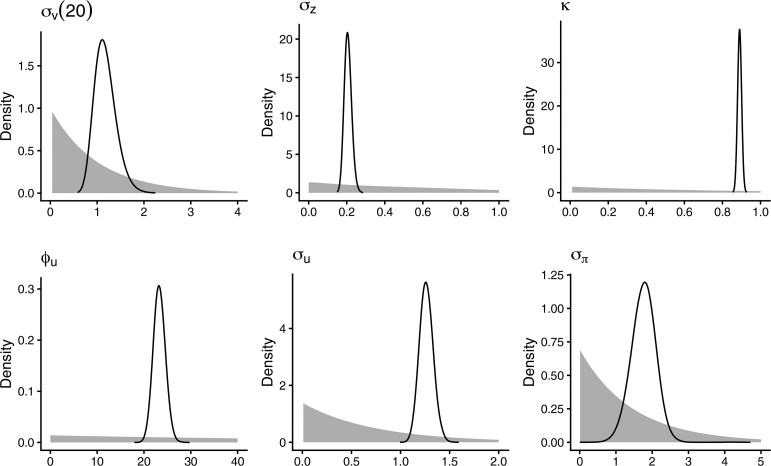
Priors and posteriors distributions for hyperparameters from the wastewater model and under-reported PAR model for Covid-19 in New Zealand. Posterior distributions are shown in black and the respective prior distributions are shaded in grey.

## Data Availability

The wastewater data that support the findings in Section 3.1 was previously available on the Government of Canada’s wastewater monitoring dashboard at https://health-infobase.canada.ca/wastewater/. Two copies of the historical data used for the analyses were merged and are both available in this paper’s github repository with the code to merge them. Laboratory confirmed weekly Covid-19 case counts for the *Toronto Public Health* Public health unit were downloaded from https://www.publichealthontario.ca/en/Data-and-Analysis/Infectious-Disease/Respiratory-Virus-Tool. These data are aggregated by epidemiological week (Sunday to Saturday), and the data used in this analysis span the period from October 4, 2020, to April 27, 2024. During this period, weekly case counts ranged from 76 to 26,855, with the 25th percentile, median, mean, and 75th percentile at 439, 1,006, 2,053, and 2,312, respectively. The wastewater data that support the findings in Section 3.2, for the new Zealand analysis, is from Covid-19 Data Repository by the Institute of Environmental Science and Research at https://github.com/ESR-NZ/covid_in_wastewater (Hewitt et al. 2022). The Covid-19 case data is from the Ministry of Health at https://github.com/minhealthnz/nz-covid-data.

## ACCESS TO DATA AND COMPUTER CODE

The code and data to reproduce all the results in the paper are provided in the github repository https://github.com/emilysomerset/wastewater_PAR_paper.

## APPENDIX A. SUPPLEMENTARY ANALYSIS: NEW ZEALAND

To align with the analysis in Watson et al. (2024), we use the total number of confirmed and probable cases across all health districts. Case counts are aggregated by epidemiological week, defined as Sunday to Saturday, covering the period from the week ending November 20, 2021, to the week ending March 25, 2023. During this period, weekly case counts ranged from 395 to 139,301, with the 25th percentile, median, mean, and 75th percentile at 9,755, 20,866, 31,719, and 49,768, respectively.

The wastewater data comes from the Aotearoa New Zealand Wastewater Covid-19 Surveillance Programme and is reported as the daily concentration of SARS-CoV-2 genome copies per liter (gc/L) of wastewater. We use wastewater observations from November 12, 2021, to March 31, 2023, totaling 10,363 measurements from 134 wastewater treatment plants. Over this 505-d period, samples were not collected on 132 d, with most non-sampled days occurring on Saturdays (50%) and Sundays (40%). Sampling times are irregular, with a median frequency of two samples per week per site. Each site contributes a median of 116 data points (IQR: 71), ranging from a minimum of 8 to a maximum of 164 data points.

Among the 10,363 wastewater samples, 1,997 (19%) are marked as undetected, indicating concentrations below the detection limit of 500 gc/L. Additionally, 892 (9%) detected samples have gc/L values exactly at the detection limit and are treated as censored at or below this threshold in subsequent analyses. The percentage of censored samples per station has a median of 30% (IQR: 30%). In 2023, the Auckland region had an estimated population of approximately 1.7 million. However, the sum of the catchment populations for sites within Auckland in the dataset is approximately 2.6 million. Upon review, we identified a site with a likely catchment population of around 300,000 instead of the recorded 1,150,000. We adjusted this site’s population estimate, assigning it a weight of 6.31% in the population-weighted wastewater signal. The site with the highest weight is Auckland East, with a catchment population of 680,000 and a weight of 16.1%.

Results from fitting the wastewater model to New Zealand’s data are shown in Fig. B3. Posterior prediction intervals for New Zealand’s Covid-19 signal are shown for both $ \bar{\mu}(t) $ in Fig. B3a and $ \bar{V}(t) $ in Fig. B3b, with the corresponding growth rates in Fig. B3c and d. We identify three distinct Omicron waves in 2022, peaking in March, July, and December. The signal accounting for station-specific deviations, seen in Fig. B3b, suggests a later onset of the first Omicron wave, with the 95% credible interval of its derivative remaining above zero from February 8, compared to January 30 in the unweighted trend. Figure B4 shows posterior prediction intervals for Covid-19 signals, $ \mu_{i}(t) $, and scaled common trends, $ \exp[\beta_{i}+V(t)] $, at 15 stations. Sites are arranged in descending order of catchment population size, from left to right and top to bottom, with the largest site in the top-left. The signal incorporating station-specific deviations aligns more closely with the data, as expected for a model covering 134 stations across an entire country. We define the wastewater signal as (2.3) for epidemic curve reconstruction. Figure B5 presents the posterior and prior distributions for the wastewater and infection count model parameters.
