# Supplementary figures and images for: Wastewater-based reproduction rates for epidemic curve reconstruction
Source: Biostatistics. 2025 Oct 17;26(1):kxaf033. doi: 10.1093/biostatistics/kxaf033 (PMC12533577; doi:10.1093/biostatistics/kxaf033)

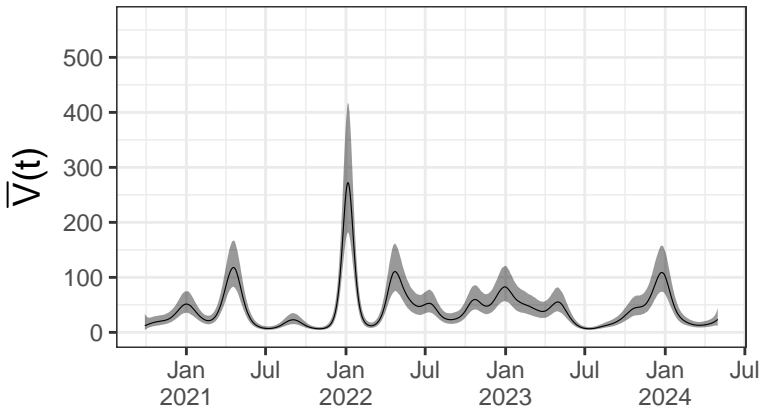

Supplement: kxaf033_Supplementary_Data [file kxaf033_supplementary_data.zip › figures/wastewater_toronto/allsignals_ON_wastewatermodel_a.pdf]

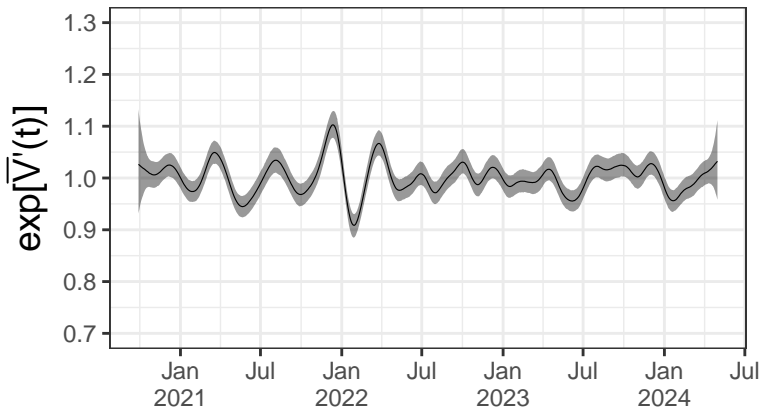

Supplement: kxaf033_Supplementary_Data [file kxaf033_supplementary_data.zip › figures/wastewater_toronto/allsignals_ON_wastewatermodel_c.pdf]

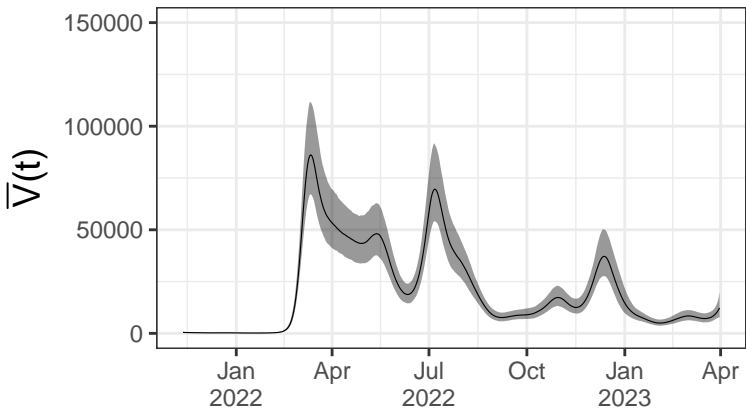

Supplement: kxaf033_Supplementary_Data [file kxaf033_supplementary_data.zip › figures/wastewater_newzealand/allsignals_NZ_wastewatermodel_a.pdf]

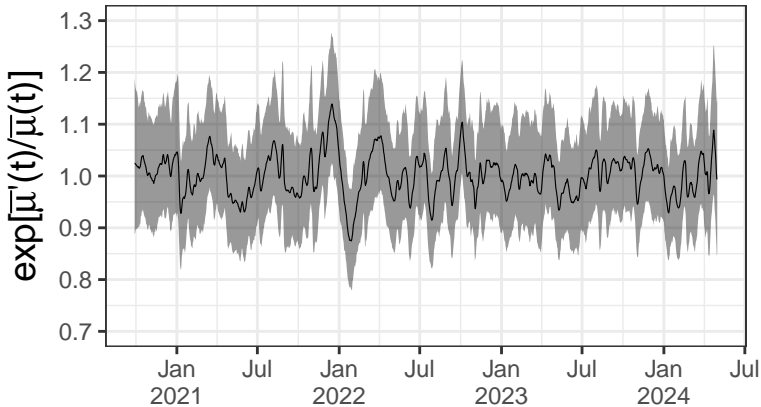

Supplement: kxaf033_Supplementary_Data [file kxaf033_supplementary_data.zip › figures/wastewater_toronto/allsignals_ON_wastewatermodel_d.pdf]

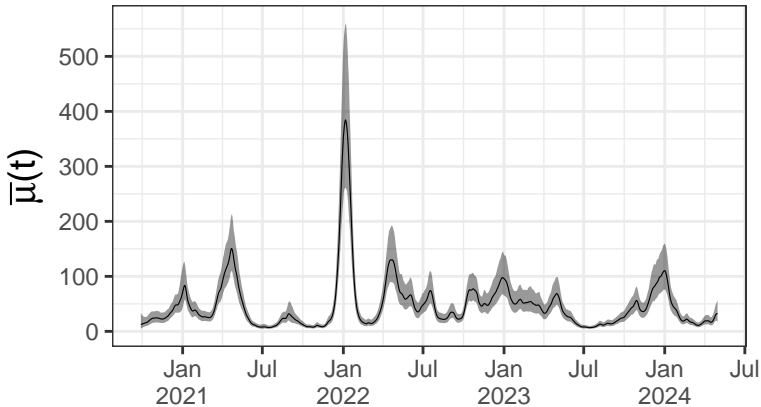

Supplement: kxaf033_Supplementary_Data [file kxaf033_supplementary_data.zip › figures/wastewater_toronto/allsignals_ON_wastewatermodel_b.pdf]

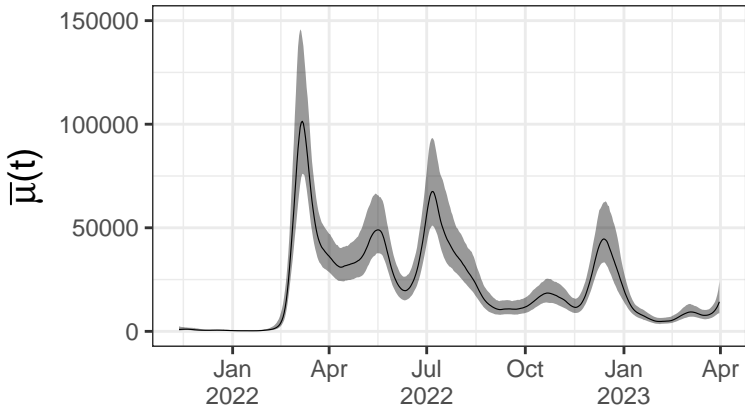

Supplement: kxaf033_Supplementary_Data [file kxaf033_supplementary_data.zip › figures/wastewater_newzealand/allsignals_NZ_wastewatermodel_b.pdf]

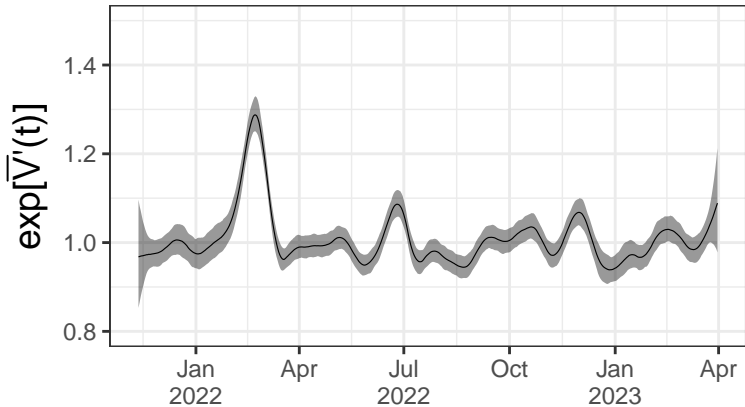

Supplement: kxaf033_Supplementary_Data [file kxaf033_supplementary_data.zip › figures/wastewater_newzealand/allsignals_NZ_wastewatermodel_c.pdf]

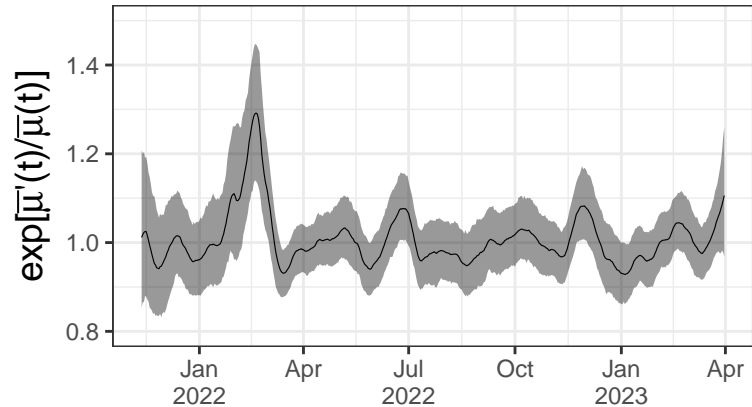

Supplement: kxaf033_Supplementary_Data [file kxaf033_supplementary_data.zip › figures/wastewater_newzealand/allsignals_NZ_wastewatermodel_d.pdf]

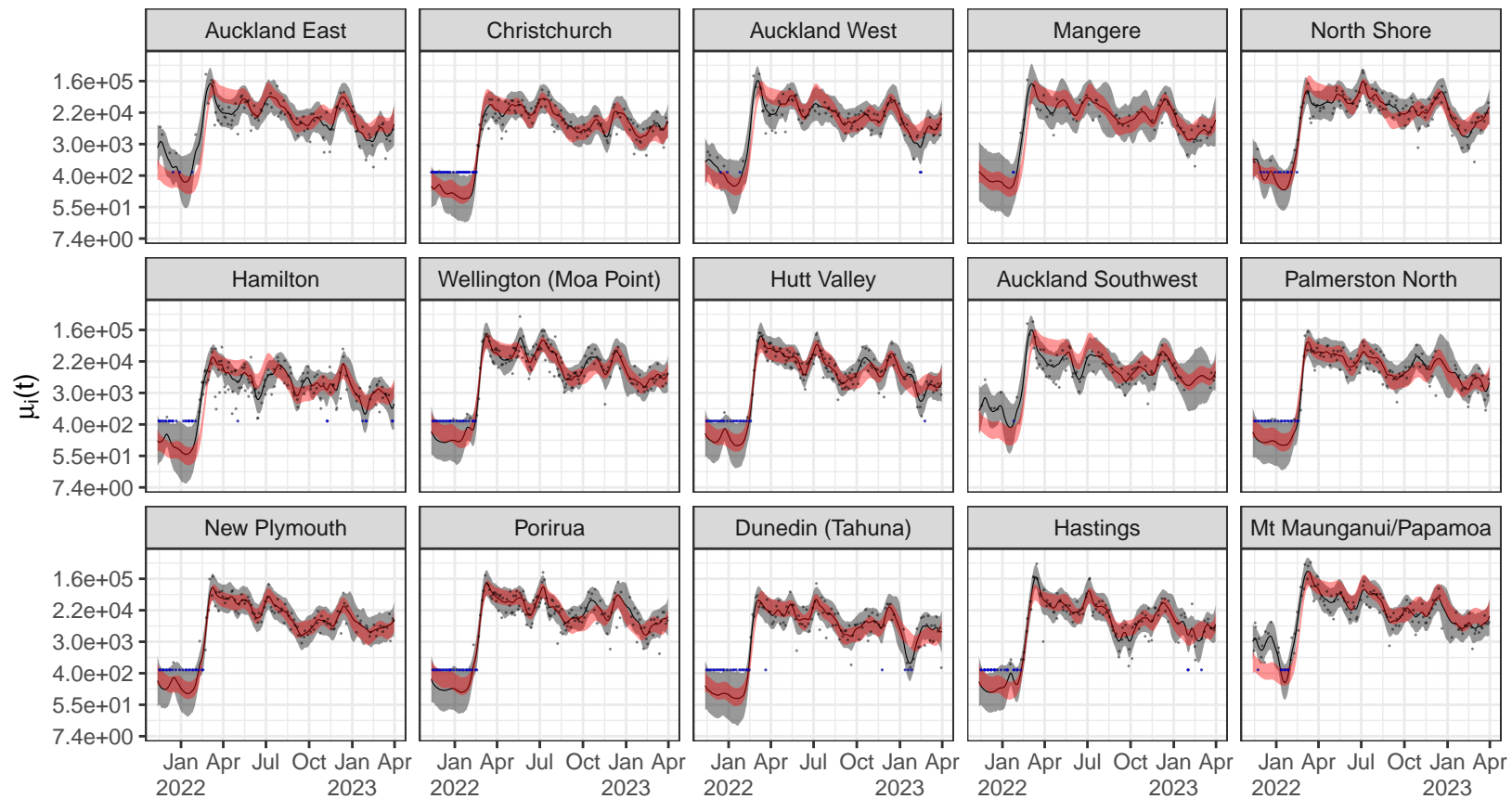

Supplement: kxaf033_Supplementary_Data [file kxaf033_supplementary_data.zip › figures/wastewater_newzealand/time_trend_fixed_AR_data.pdf]

$\sigma_v(20)$ 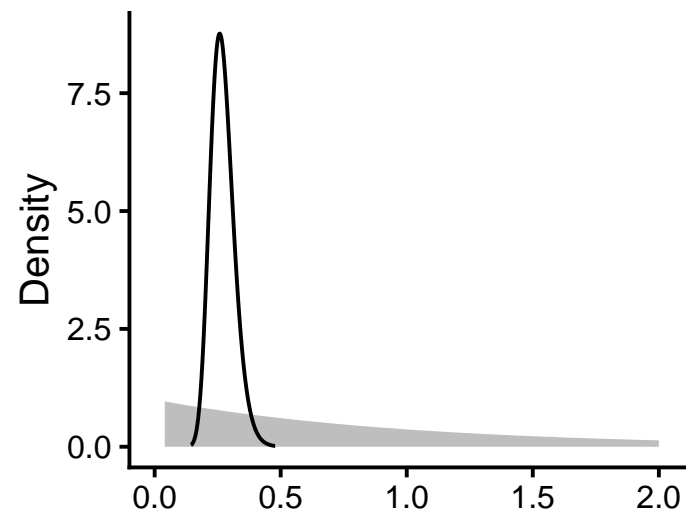 $\sigma_z$ 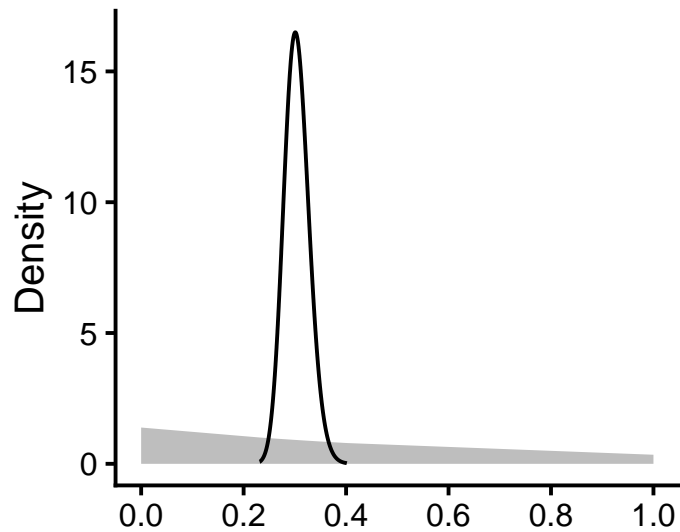 $\kappa$ 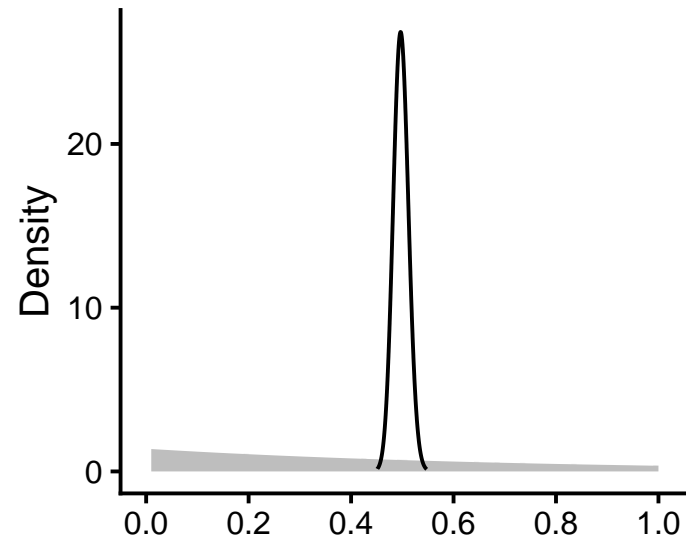 $\phi_u$ 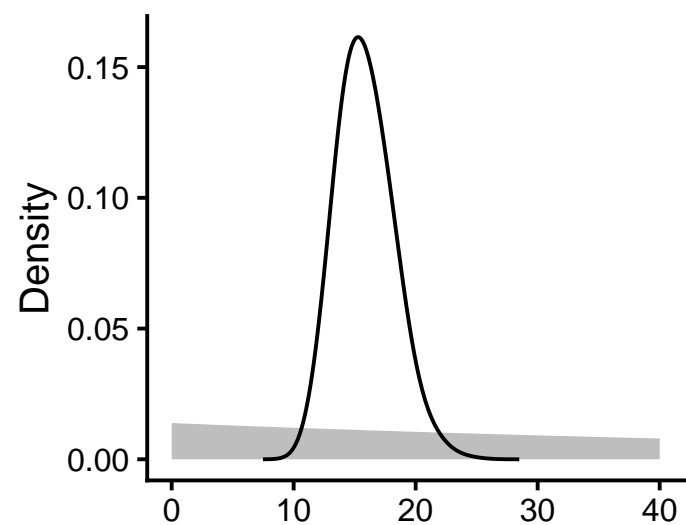 $\sigma_u$ 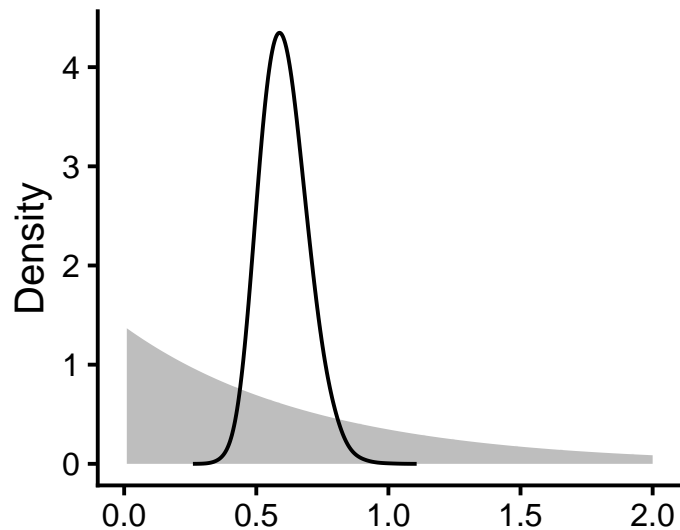 $\sigma_\pi$ 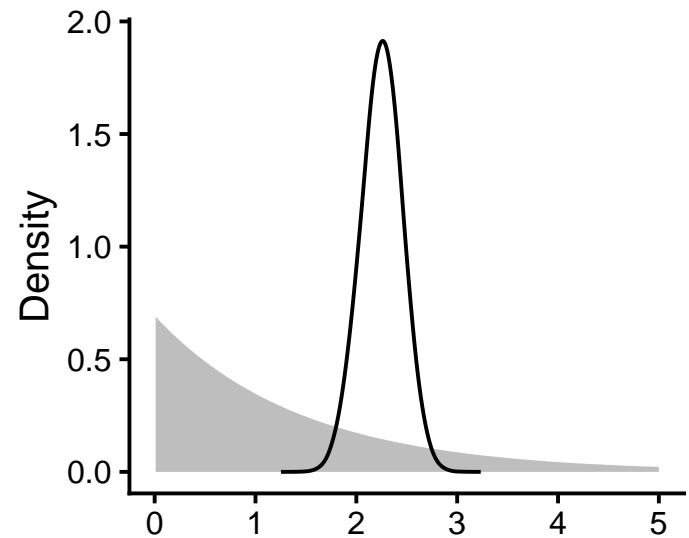

Supplement: kxaf033_Supplementary_Data [file kxaf033_supplementary_data.zip › figures/hyperparameters_toronto/hyperparameters_ON.pdf]

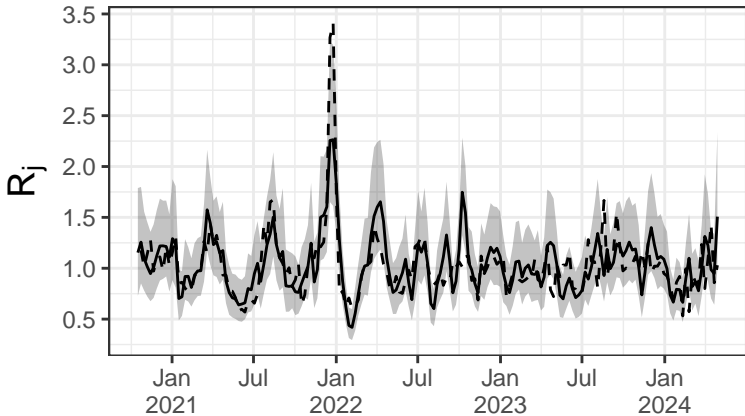

Supplement: kxaf033_Supplementary_Data [file kxaf033_supplementary_data.zip › figures/epidemic_toronto/epidemic_model_toronto_a.pdf]

$\sigma_v(20)$ 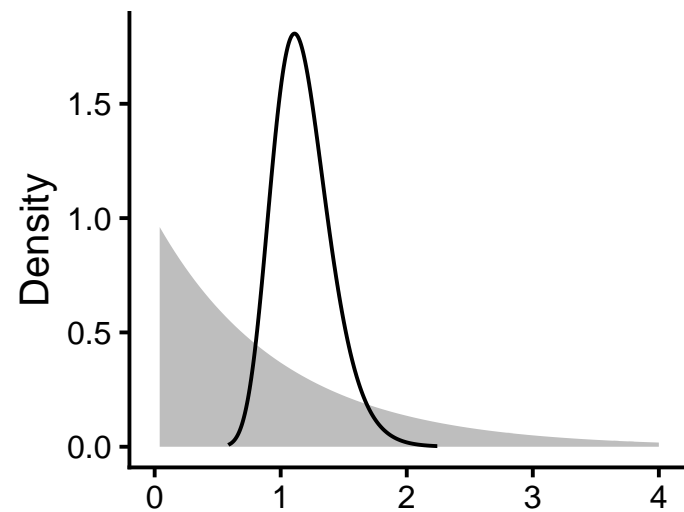 $\sigma_z$ 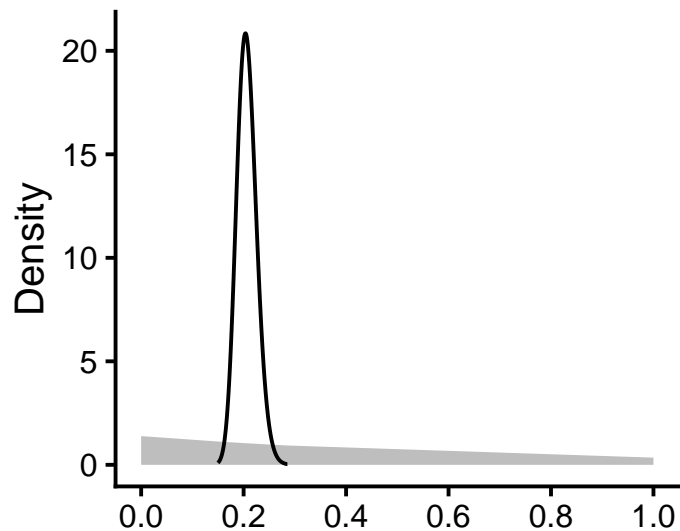 $\kappa$ 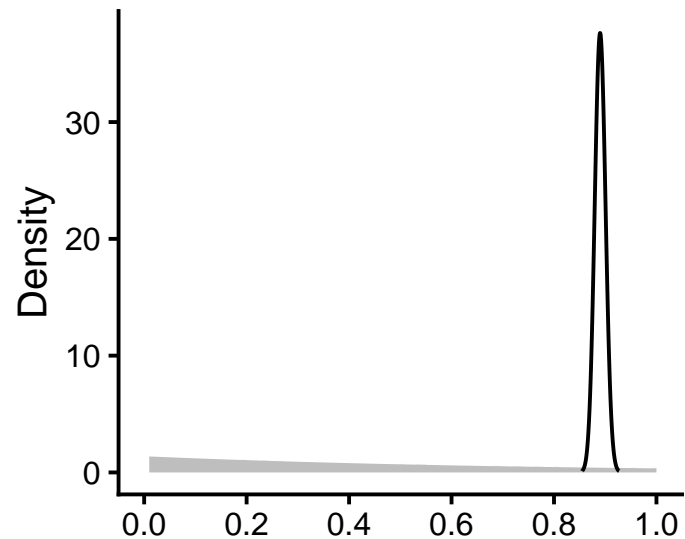 $\phi_u$ 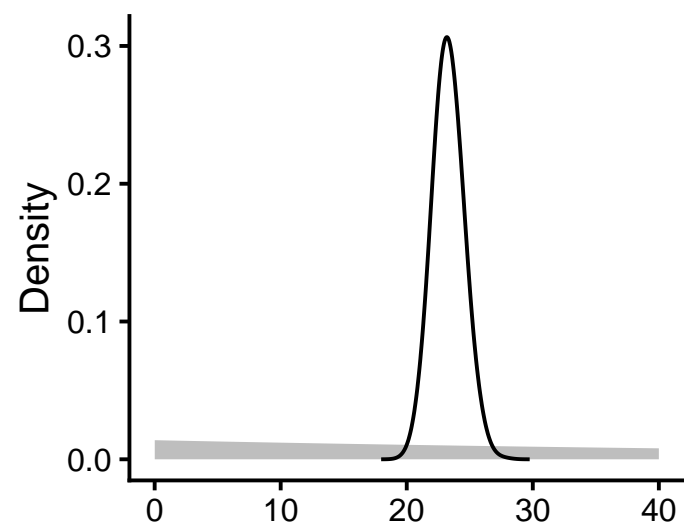 $\sigma_u$ 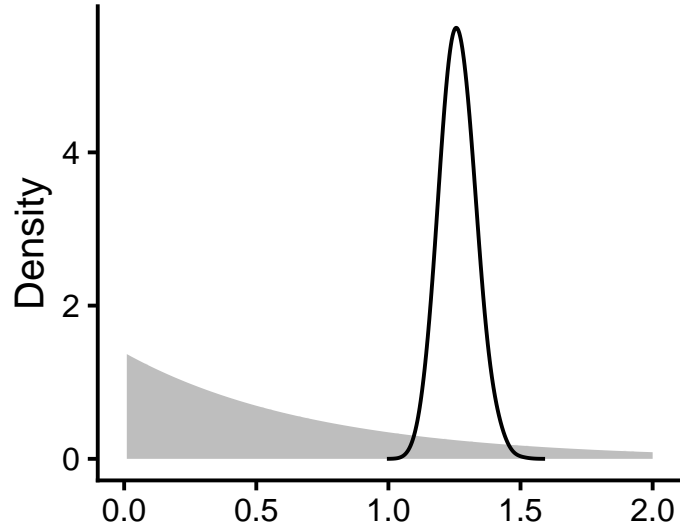 $\sigma_\pi$ 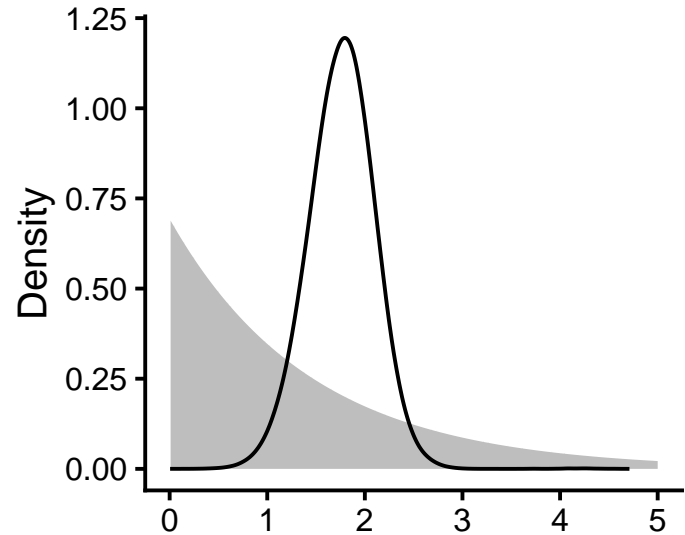

Supplement: kxaf033_Supplementary_Data [file kxaf033_supplementary_data.zip › figures/hyperparameters_NZ/hyperparameters_NZ.pdf]

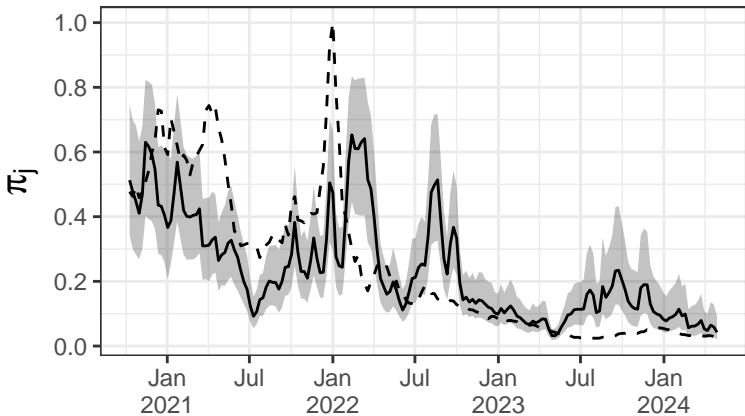

Supplement: kxaf033_Supplementary_Data [file kxaf033_supplementary_data.zip › figures/epidemic_toronto/epidemic_model_toronto_b.pdf]

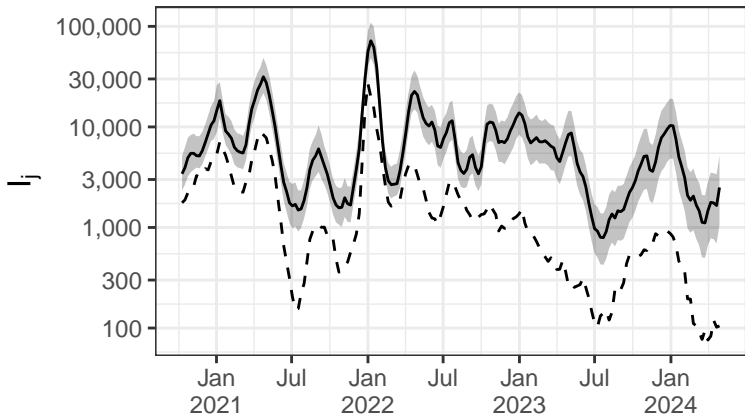

Supplement: kxaf033_Supplementary_Data [file kxaf033_supplementary_data.zip › figures/epidemic_toronto/epidemic_model_toronto_c.pdf]

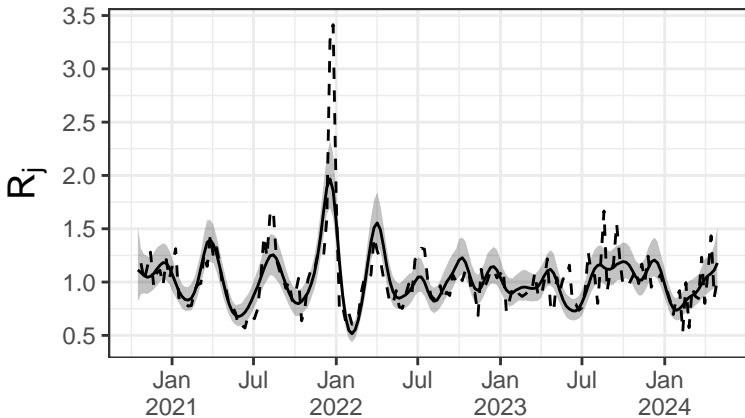

Supplement: kxaf033_Supplementary_Data [file kxaf033_supplementary_data.zip › figures/epidemic_toronto/epidemic_model_toronto_common_a.pdf]

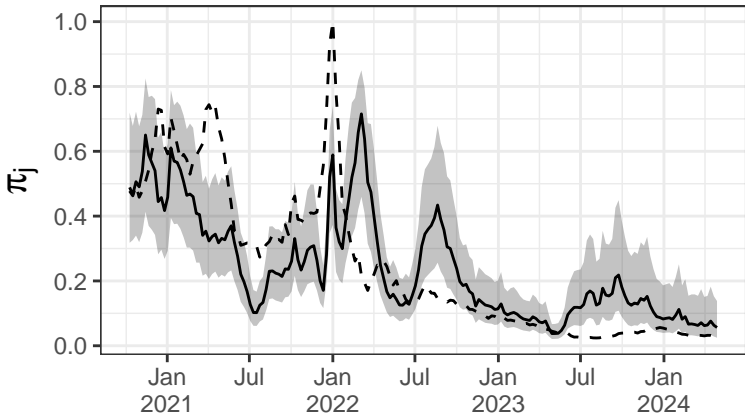

Supplement: kxaf033_Supplementary_Data [file kxaf033_supplementary_data.zip › figures/epidemic_toronto/epidemic_model_toronto_common_b.pdf]

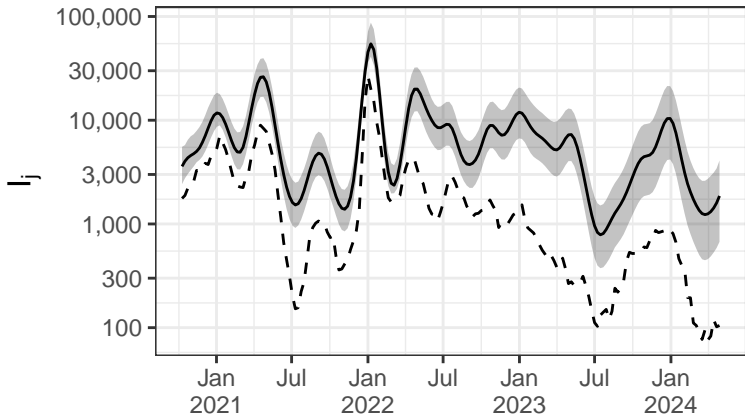

Supplement: kxaf033_Supplementary_Data [file kxaf033_supplementary_data.zip › figures/epidemic_toronto/epidemic_model_toronto_common_c.pdf]

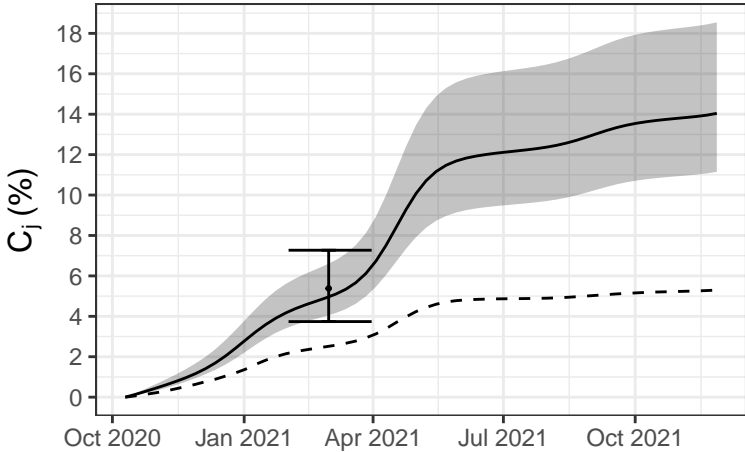

Supplement: kxaf033_Supplementary_Data [file kxaf033_supplementary_data.zip › figures/epidemic_toronto/epidemic_model_toronto_common_d.pdf]

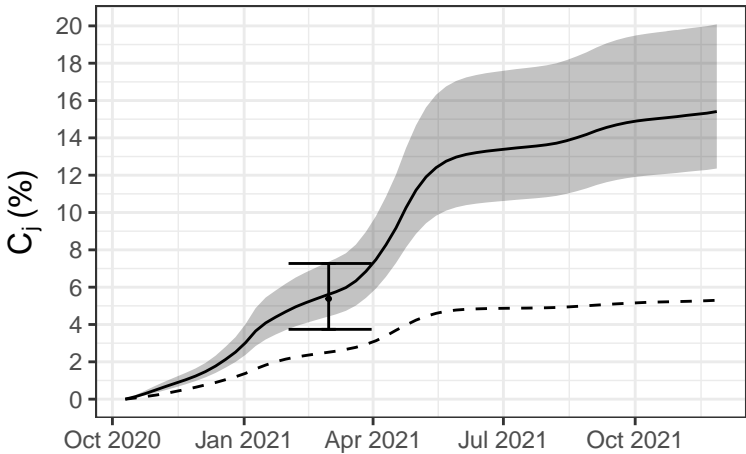

Supplement: kxaf033_Supplementary_Data [file kxaf033_supplementary_data.zip › figures/epidemic_toronto/epidemic_model_toronto_d.pdf]

COVID-19 case counts

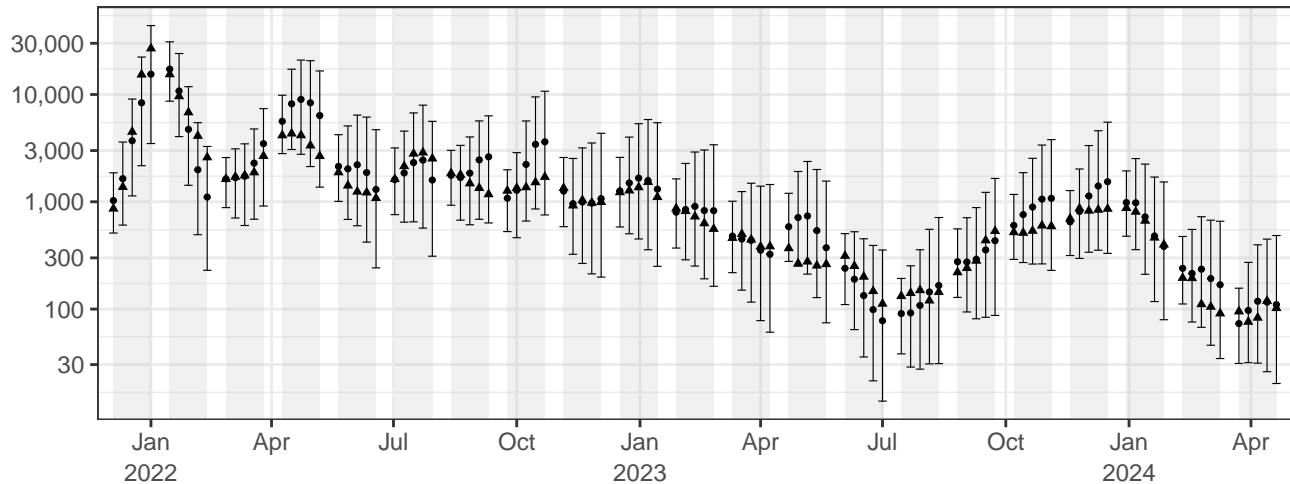

Supplement: kxaf033_Supplementary_Data [file kxaf033_supplementary_data.zip › figures/epidemic_toronto/epidemic_model_validation_toronto.pdf]

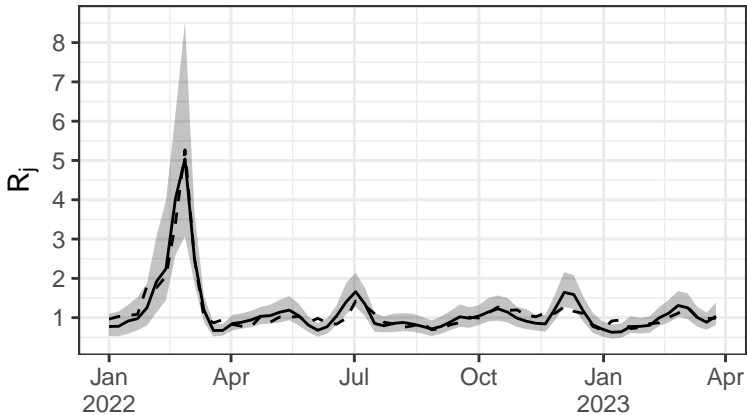

Supplement: kxaf033_Supplementary_Data [file kxaf033_supplementary_data.zip › figures/epidemic_newzealand/epidemic_model_NZ_a.pdf]

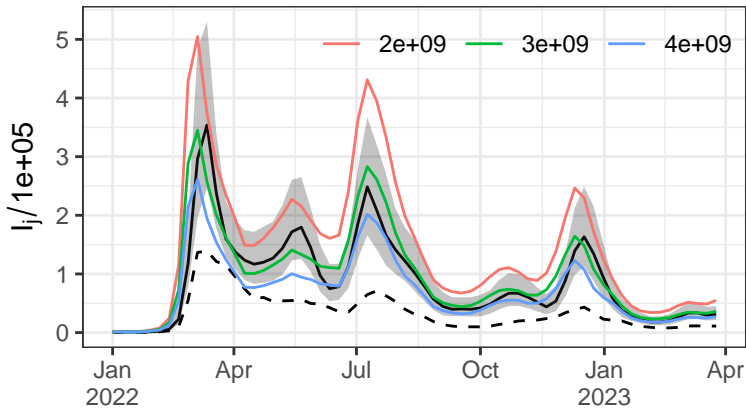

Supplement: kxaf033_Supplementary_Data [file kxaf033_supplementary_data.zip › figures/epidemic_newzealand/epidemic_model_NZ_b.pdf]

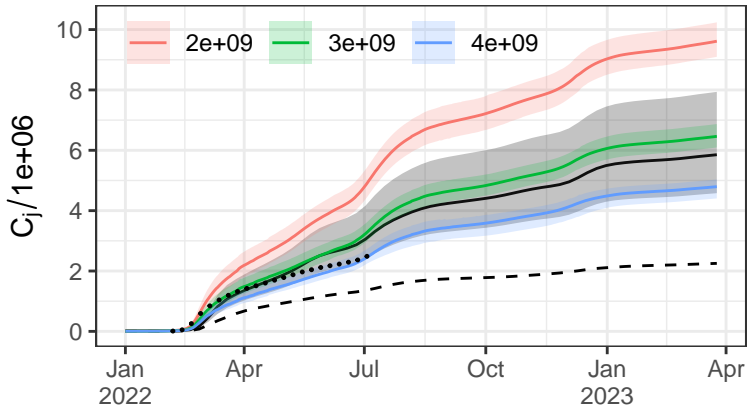

Supplement: kxaf033_Supplementary_Data [file kxaf033_supplementary_data.zip › figures/epidemic_newzealand/epidemic_model_NZ_c.pdf]

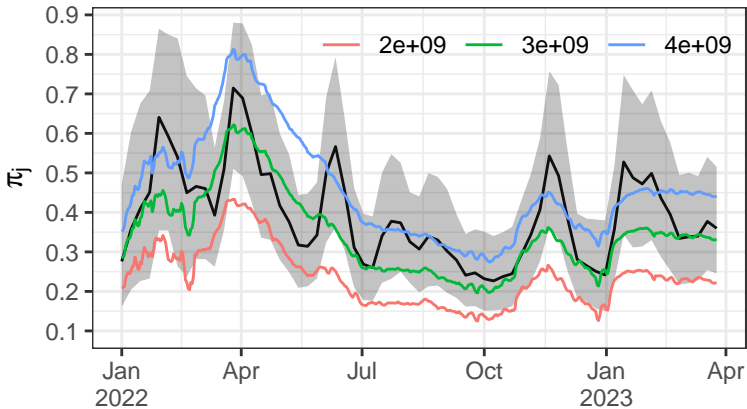

Supplement: kxaf033_Supplementary_Data [file kxaf033_supplementary_data.zip › figures/epidemic_newzealand/epidemic_model_NZ_d.pdf]
